# Supplementary material for: Multi-omics analyses reveal that the gut microbiome and its metabolites promote milk fat synthesis in Zhongdian yak cows
Source: PeerJ. 2022 Dec 2;10:e14444. doi: 10.7717/peerj.14444 (PMC9744170; doi:10.7717/peerj.14444)
Supplement: Supplemental Information 13 [file peerj-10-14444-s013.zip › Web_Report/index.html]

BMK211021-AP383-ZX01-0201|西南林业大学10个宏基因组12个代谢组建库测序分析

BMK211021-AP383-ZX01-0201|西南林业大学10个宏基因组12个代谢组建库测序分析

## BMK211021-AP383-ZX01-0201|西南林业大学10个宏基因组12个代谢组建库测序分析

### 摘要

代谢物是生物体表型的基础，能帮助更直观有效地了解生物学过程以及其机理。基于对代谢物的定性定量分析，代谢组学可以用于研究代谢途径或代谢网络的解析，不同生物个体的代谢组学表型现象，不同疾病、药物等物理、化学或病原生物刺激后代谢产物的应答机制，以及食品、药物等安全评价。 代谢组学分析主要目的是从生物样本中检测并筛选出具有重要生物学意义和统计显著差异的代谢物，并以此为基础阐明生物体的代谢过程和变化机制。代谢组学分析包括代谢组学实验及数据分析两大部分，其中数据分析主要包括差异代谢物筛选及代谢途径解析。基于实验设计、样本的采集及处理、代谢物提取以及代谢物的检测分析获取的代谢组数据，可以进行代谢物的鉴定与样本数据的质控分析，并筛选出一些有差异的代谢物，从而对样本的代谢物进行相关的功能预测和分析。 本项目基于LC-QTOF平台，对12个样本进行代谢组定性和定量分析，共检测到了2,400个代谢物。 样本分组信息如下：

表1 样本信息表

| Sample | Group |
| --- | --- |
| L1 | L |
| L5 | L |
| L4 | L |
| L2 | L |
| L6 | L |
| L3 | L |
| H5 | H |
| H4 | H |
| H2 | H |
| H6 | H |
| H1 | H |
| H3 | H |

### 1 代谢组主要分析内容

以下分析内容均由百迈客云平台BMKCloud（www.biocloud.net）的生物信息学分析流程分析所得。

1.数据评估

（1）主成分分析

（2）所有样本聚类分析

（3）重复相关性评估

2.样本分组数据分析

（1）差异分组的主成分分析

（2）差异分组的差异倍数分析

（3）差异分组的正交偏最小二乘法判别分析

（4）各分组的差异代谢物筛选及绘图

（5）差异代谢物KEGG通路注释及富集分析

（6）ROC分析（绝对定量且样本较多时）

图1 代谢组分析流程图

### 2 非靶向代谢组分析结果

#### 2.1 实验流程

##### 2.1.1 实验试剂与仪器

表2 实验试剂列表

| 名称 | CAS | 纯度 | 品牌 |
| --- | --- | --- | --- |
| 甲醇（Methanol） | 67-56-1 | LC-MS级 | CNW Technologies |
| 乙腈（Acetonitrile） | 75-05-8 | LC-MS级 | CNW Technologies |
| L-2-氯苯丙氨酸（2-Chloro-L-phenylalanine） | 103616-89-3 | ≥98% | 上海阿拉丁 |
| 甲酸(Formic acid) | 64-18-6 | LC-MS级 | TCI |

表3 实验仪器列表

| 仪器 | 型号 | 品牌 |
| --- | --- | --- |
| 超高效液相 | Waters UPLC Acquity I-Class PLUS | Waters |
| 高分辨质谱 | Waters UPLC Xevo G2-XS QTOF | Waters |
| 色谱柱 | Acquity UPLC HSS T3 1.8um 2.1\*100mm | Waters |

##### 2.1.2 样品提取

主要处理流程如下：

（1）移取100μL样本至1.5ml EP管中，加入300μL甲醇，再加入20μL内标，涡旋混匀30秒；

（2）超声10min（冰水浴）；

（3）零下20℃ 静置一小时；

（4）将样本4℃，13000rpm离心15min；

（5）小心地取出200μL上清于2mL进样瓶，每个样本各取20μL混合成QC样本，再取200μL上机检测。

##### 2.1.3 上机检测

用于代谢组学分析的液质联用系统由沃特世Acquity I-Class PLUS超高效液相串联沃特世Xevo G2-XS QTof高分辨质谱仪组成所使用色谱柱为购自沃特世的Acquity UPLC HSS T3 色谱柱（1.8um 2.1\*100mm）

正离子模式：流动相A：0.1%甲酸水溶液；流动相B：0.1%甲酸乙腈

负离子模式：流动相A：0.1%甲酸水溶液；流动相B：0.1%甲酸乙腈

进样体积1ul

表4 液相色谱流动相条件

| 时间(min) | 流速(μL/min) | A%水（25mM醋酸铵及25mM氨水） | B%乙腈 |
| --- | --- | --- | --- |
| 0 | 400 | 98 | 2 |
| 0.25 | 400 | 98 | 2 |
| 10.00 | 400 | 2 | 98 |
| 13.00 | 400 | 2 | 98 |
| 13.10 | 400 | 98 | 2 |
| 15.00 | 400 | 98 | 2 |

沃特世Xevo G2-XS QTof高分辨质谱仪能够在采集软件（MassLynx V4.2,Waters）控制下的MSe模式进行一级、二级质谱数据采集。在每个数据采集循环中，能够同时对低碰撞能量及高碰撞能量进行双通道数据采集。低碰撞能量2V，高碰撞能量区间为10~40V，扫描频率为0.2秒一张质谱图。ESI离子源参数如下：毛细管电压：2000V（正离子模式）或-1500V（负离子模式）；锥孔电压：30V；离子源温度：150℃；脱溶剂气温度500℃；反吹气流速：50L/h；脱溶剂气流速：800L/h。

常见问题

- 什么是正负离子模式？结果以哪个为准？

  在ESI电离源下，由于代谢物的结构性质不同，会生成正离子或负离子，仪器分别用正负离子模式来检测不同电荷的离子，两种模式下的结果都是可信的。当两种模式下的结果差异比较大时（有数量级差异），可以选择峰面积大的离子模式；如果差异不大（没有数量级的差异），正离子模式的打分值减负离子模式的打分值>-1，选负离子模式。

##### 2.1.4 代谢物定量

使用MassLynx V4.2采集的原始数据通过Progenesis QI软件做峰提取、峰对齐等数据处理操作，基于Progenesis QI软件在线METLIN数据库及百迈客自建库进行鉴定，同时进行理论碎片识别，质量数偏差均在100ppm以内。

分析前先对数据进行了归一化处理，采取的方式是总峰面积归一化即每个样本的每个代谢物除以该样本总的峰面积，处理后的代谢物定量信息如下：

表5 代谢物数量统计表

| ID | name | H1 | H2 | H3 | H4 | H5 | H6 | L2 | L6 | L1 | L4 | L5 | L3 |
| --- | --- | --- | --- | --- | --- | --- | --- | --- | --- | --- | --- | --- | --- |
| pos\_1 | D-erythro-Dihydrosphingosine | 0.00014 | 0.00028 | 0.00022 | 0.00025 | 0.00017 | 0.0002 | 0.00014 | 0.00014 | 0.00013 | 0.00013 | 0.00016 | 0.00012 |
| pos\_10 | Deoxycholic acid | 0.027 | 0.023 | 0.067 | 0.06 | 0.075 | 0.058 | 0.049 | 0.044 | 0.068 | 0.054 | 0.057 | 0.052 |
| pos\_100 | Dihydroscoparin | 3.39668072962394e-05 | 4.77892415245145e-05 | 7.52120583590671e-05 | 0.00022 | 8.73793923402068e-05 | 2.03705463933448e-05 | 4.8823197771671e-05 | 3.2263567000144e-05 | 6.93457125590401e-05 | 4.25746516619048e-05 | 0.00013 | 9.17680508017233e-05 |
| pos\_1000 | Lansioside C | 0.00092 | 0.00046 | 0.00041 | 0.0004 | 0.00048 | 0.00049 | 0.00051 | 0.00049 | 0.00046 | 0.00044 | 0.00047 | 0.00047 |
| pos\_1001 | Hexylamine | 4.01384358402482e-12 | 3.9576159497502e-12 | 6.57739001429416e-07 | 0 | 0 | 0 | 2.40099525812225e-06 | 2.22730488235693e-06 | 0 | 4.64508343563833e-09 | 1.20430046582174e-06 | 0.00049 |
| pos\_1002 | Ginsenoyne I | 4.53350425687984e-06 | 9.75242210291292e-06 | 8.47109894285107e-06 | 1.02142773637819e-05 | 1.21264494948e-05 | 9.52843959345179e-06 | 8.35859896511495e-06 | 9.45917829860012e-06 | 1.02390284793562e-05 | 9.27516222460088e-06 | 7.36279430579978e-06 | 1.0737592159788e-05 |
| pos\_1003 | Testolactone | 0.00026 | 0.0002 | 0.00018 | 0.00017 | 0.00018 | 0.0002 | 0.00021 | 0.0002 | 0.00021 | 0.00018 | 0.00019 | 0.00019 |
| pos\_1004 | DG(20:4(5Z,8Z,11Z,14Z)/22:5(4Z,7Z,10Z,13Z,16Z)/0:0) | 0.00033 | 0.00032 | 0.0004 | 0.00021 | 0.00018 | 0.00026 | 0.00016 | 0.00027 | 0.00039 | 0.00019 | 0.00029 | 0.00041 |
| pos\_1005 | Bovinic acid | 7.27384813662508e-05 | 5.06632478351181e-05 | 4.66893124317539e-05 | 4.74674269697067e-05 | 3.34830463533819e-05 | 3.23635637828467e-05 | 3.76905549919153e-05 | 4.30996677093678e-05 | 3.10740657141884e-05 | 3.51713540738406e-05 | 3.54024016204916e-05 | 3.0586890091983e-05 |

注：第一列表示代谢物的代号；  
第二列表示代谢定性的物质名称,若为空则表示未定性；  
第三列到最后表示各个样本的归一化后的代谢物丰度。

metabolites\_exp.xls

常见问题

- 代谢物定量是否为绝对定量？

  这个取决于技术手段，如果是非靶代谢组，则用的是峰面积即相对定量，就不是绝对定量；如果是靶标、或者部分高通量靶标产品则为绝对定量。建议基于研究目的了解清楚是否需要绝对定量从而选择合适的产品。

- 归一化方式如何选择？

  目前预设的有峰面积归一化和内标归一化方式，一般而言如果是非靶LC的建议使用峰面积归一化，如果是非靶GC的建议使用内标归一化。也可以自己先对数据采取其他方式进行预处理，然后在BMK代谢分析平台提交页面选择的时候选不归一化。

- 结题报告中的代谢物定量数值为何很小？

  这个是由于数据归一化方式导致的，对于非靶项目如果采取了峰面积归一化即每个代谢物的峰面积会除以该样本所有代谢物的总峰面积，这样进行处理后的数值就会变的很小了。

- 样本没有重复是否可以分析？

  无法使用，因为目前流程使用的差异代谢物筛选方法是t检验和OPLS-DA分析，这两个分析都需要有重复。

#### 2.2 数据评估

##### 2.2.1 主成分分析（PCA）

主成分分析（Principal Component Analysis，PCA）是一种无监督模式识别的多维数据统计分析方法，通过正交变换将一组可能存在相关性的变量转换为一组线性不相关的变量，转换后的这组变量叫主成分。这个分析方法常用来研究如何通过少数几个主成分来揭示多个变量间的内部结构，即从原始变量中导出少数几个主成分，使它们尽可能多地保留原始变量的信息，且彼此间互不相关，通常数学上的处理就是将原来多个指标作线性组合，作为新的综合指标。通过对样本（包括质控样品）进行主成分分析，以便初步了解各组样本之间的总体代谢差异和组内样本之间的变异度大小。

所有样本的主成分分析结果如下图：

- All\_pca
- All\_pca3D

图2 所有样本PCA分析

注：其中X轴表示第一主成分，Y轴表示第二主成分。

##### 2.2.2 聚类热图分析

聚类分析（Cluster Analysis）是根据“物以类聚”的道理，对样品或指标进行分类的一种多元统计分析方法，通常能简单、直观的观察数据特征。

对所有样品进行聚类热图分析，其结果如下图：

图3 所有样本聚类图

##### 2.2.3 重复相关性评估

通过样品之间的相关性分析可以评估组内样品之间的生物学重复。同时组内样品相对组间样品的相关系数越高，获得的差异代谢物越可靠。将斯皮尔曼等级相关系数r（Spearman Rank Correlation）作为生物学重复相关性的评估指标。r2越接近1，说明两个重复样品相关性越强。结果见下图：

图4 样品间相关性图

#### 2.3 代谢物注释

##### 2.3.1 KEGG数据库注释

生物体中的复杂代谢反应及其调控并不单独进行，往往由不同基因和蛋白质形成复杂的通路和网络，它们的相互影响和相互调控最终导致代谢组发生系统性的改变。对这些代谢和调控通路的分析可以更全面，更系统的了解实验条件改变导致的生物学过程的改变，性状或疾病的发生机理和药物作用机制等生物学问题。

KEGG（Kyoto Encyclopedia of Genes and Genomes）数据库有助于研究者把基因、表达信息以及代谢物含量作为一个整体网络进行研究。作为有关Pathway的主要公共数据库，KEGG提供的整合代谢途径 (pathway)查询，包括碳水化合物、核苷、氨基酸等的代谢及有机物的生物降解，不仅提供了所有可能的代谢途径，而且对催化各步反应的酶进行了全面的注解，包含有氨基酸序列、PDB库的链接等等，是进行生物体内代谢分析、代谢网络研究的强有力工具。

差异代谢物在生物体内相互作用，形成不同的通路。利用KEGG数据库对所有鉴定到的代谢物进行注释，选取KO pathway level2条目注释最多的top20注释信息绘图以及注释表格如下：

图5 KEGG数据库

KEGG数据库注释结果以及分类结果

1. meta\_kegg\_anno.xls
2. meta\_kegg\_anno\_categroy.xls

##### 2.3.2 HMDB数据库注释

HMDB（Human Metabolome Database） 是一个免费提供的电子数据库，其中包含有关人体中发现的小分子代谢物的详细信息。广泛用于代谢组学、临床化学、生物标志物发现的研究中。该数据库旨在包含或链接三种数据：1) 化学数据，2) 临床数据，以及 3) 分子生物学/生物化学数据。选取注释信息中注释最多的top 20 class绘图结果以及注释表格如下：

图6 HMDB数据库分类汇总

HMDB数据库注释结果以及分类结果

1. meta\_hmdb\_anno.xls
2. meta\_hmdb\_anno\_categroy.xls

##### 2.3.3 Lipidmaps数据库注释

LIPID MAPS（脂质代谢物和通路策略）是一个经典的脂质数据库，旨在成为脂质组学资源的门户。LIPID MAPS 于 2003 年由 NIH创建，提供对脂质命名法、数据库、工具、协议、标准、教程、会议、出版物和其他资源的访问，并为国际脂质研究社区提供服务。该数据库率先对生物脂质进行了分类，将它们分为八个大类。 LIPID MAPS为脂质质谱分析提供标准化方法，已成为对脂质代谢研究以及脂质组学领域的快速发展和标准化的日益重视的证据。选取注释信息中注释最多的top 20 CATEGORY绘图结果以及注释表格如下：

图7 LIPID MAPS数据库分类汇总

Lipidmaps数据库注释结果以及分类结果

1. meta\_lipidmaps\_anno.xls
2. meta\_lipidmaps\_anno\_categroy.xls

常见问题

- 代谢物是如何进行数据库注释的？

  目前采取策略是直接用代谢物名称在各数据库中进行匹配，所以依赖于名称的规范性。

#### 2.4 样本分组数据分析

##### 2.4.1 各组主成分分析

在进行差异分析之前，先对各个差异分组进行PCA分析，结果如下：

- H\_vs\_L\_pca

图8 主成分分析图

差异分组的PCA三维图如下：

- H\_vs\_L\_pca3D

图9 主成分分析三维图

##### 2.4.2 差异倍数分析

对所检测到的代谢物进行定性和定量分析后，可先比较在各分组中代谢物定量信息发生的差异倍数变化。下图为各分组比较中进行Generalized log transformation（R语言LMGene包）处理后，将上下调logFC各前10的代谢物结果展示如下：

- H\_vs\_L\_Top\_20\_FC\_change

图10 差异倍数柱图

注：各柱子的标签表示代谢物ID

##### 2.4.3 差异分组的正交偏最小二乘法判别分析（OPLS-DA）

代谢组学数据具有高维（检测出代谢物种类多），小样本（检测样本量偏少）的特性，在这些变量中既包含与分类变量相关的差异变量，也包含大量互相之间可能存在关联的无差异变量。这导致如果我们使用PCA模型或PLS模型进行分析，由于相关变量的影响，差异变量会分散到更多的主成分上，无法进行更好的可视化和后续分析。所以我们采用正交偏最小二乘法-判别分析（orthogonal projections to latent structures- discriminant analysis, OPLS-DA）的统计方法对结果进行分析。通过OPLS-DA分析，我们可以过滤掉代谢物中，与分类变量不相关的正交变量，并对非正交变量和正交变量分别分析，从而获取更加可靠的代谢物的组间差异与实验组的相关程度信息。本项目主要利用R（3.3.2）包ropls进行OPLS-DA模型计算。

评价模型的预测参数有R2X，R2Y和Q2，其中R2X和R2Y分别表示所建模型对X和Y矩阵的解释率，其中X矩阵为模型输入即代谢物定量矩阵，Y矩阵为模型输出即样本分组矩阵，Q2表示模型的预测能力即所建模型能否通过代谢表达量区分正确的样本分组，指标的中R2Y和Q2越接近于1时表示模型越稳定可靠，即可以用此模型筛选差异代谢物。一般来说Q2>0.5时可认为是有效的模型，Q2>0.9时为出色的模型。各组模型得分结果如下：

- H\_vs\_L\_OPLS\_DA

图11 OPLS-DA得分图

为检查OPLS-DA模型的可靠性，需要进行排列验证，结果如下：

- H\_vs\_L\_OPLS\_DA\_permutation

图12 OPLS-DA模型验证图

注：图中横轴表示与原模型的相似度，纵轴表示R2Y或者Q2的取值（其中在横坐标取1的R2Y和Q2为原模型的值），蓝点和红点分别代表Y置换后模型的R2Y和Q2，虚线为拟合的回归线。若R2Y和Q2均小于原始模型的R2Y和Q2，即图左边（置换检验）所有点都比横坐标为1处的点（原模型）的位置低则说明模型有意义，可根据变量投影重要度（Variable Importance in the Projection, VIP）分析筛选差异代谢物。若出现原模型R2Y和Q2数值本身较低且排列检验结果不理想时，此时可不考虑将VIP作为差异代谢物的筛选条件

##### 2.4.4 差异代谢物筛选

对有生物学重复的,采取将差异倍数、t检验的P值和OPLS-DA模型的VIP值相结合的方法来筛选差异代谢物，筛选的标准为FC>1、P value<0.05和VIP>1（注：此处FC的书写方式是按照上调的表示方式，实际筛选也是包含下调的，比如FC阈值是2的时候表示筛选FC>2即上调的同时也筛选FC<1/2即下调，如果阈值是1则筛选差异时相当于不考虑FC），结果如下：

表6 差异代谢物筛选结果部分展示

| ID | name | H5 | H4 | H2 | H6 | H1 | H3 | L1 | L5 | L4 | L2 | L6 | L3 | H\_Mean | L\_Mean | Fold\_change | log2FC | Pvalue | VIP | regulated |
| --- | --- | --- | --- | --- | --- | --- | --- | --- | --- | --- | --- | --- | --- | --- | --- | --- | --- | --- | --- | --- |
| pos\_1 | D-erythro-Dihydrosphingosine | 0.00017 | 0.00025 | 0.00028 | 0.0002 | 0.00014 | 0.00022 | 0.00013 | 0.00016 | 0.00013 | 0.00014 | 0.00014 | 0.00012 | 0.00021 | 0.00014 | 0.65 | -0.6 | 0.016 | 2.29 | down |
| pos\_1073 | Neolinustatin | 8.65184137415296e-06 | 7.88622271234797e-06 | 9.85186204859414e-06 | 8.04562355504655e-06 | 5.96503890331222e-06 | 6.88259674573533e-06 | 1.27405048068724e-05 | 9.8469071217803e-06 | 8.4786020981128e-06 | 1.11270187582944e-05 | 9.08477593083803e-06 | 1.46742998177055e-05 | 7.88053088986486e-06 | 1.09920180889339e-05 | 1.39 | 0.47 | 0.023 | 2.29 | up |
| pos\_108 | RHOIFOLIN | 9.23534477713109e-06 | 2.0370241796344e-06 | 1.82688113090293e-05 | 1.09351704987591e-05 | 3.78775372116092e-06 | 3.57014084103671e-05 | 1.71236608976507e-05 | 4.17430306845687e-05 | 3.84908284112322e-05 | 8.49582130333884e-05 | 6.2976908725647e-05 | 7.69590820373692e-05 | 1.33275854826803e-05 | 5.37086206316427e-05 | 4.03 | 2.41 | 0.01 | 2.52 | up |
| pos\_1141 | Isovalerylglutamic acid | 2.03288102216096e-05 | 1.77626953626292e-05 | 2.5607171467383e-05 | 1.50831721446601e-05 | 1.72535810088484e-05 | 8.31919499750707e-06 | 3.6666681758369e-05 | 3.35311177847392e-05 | 2.53988822599635e-05 | 4.73858259215339e-05 | 3.1145888413144e-05 | 1.32588457165065e-05 | 1.73924375337729e-05 | 3.12312069757094e-05 | 1.80 | 0.82 | 0.031 | 2.17 | up |
| pos\_1143 | PE(15:0/20:2(11Z,14Z)) | 6.36832831399463e-06 | 1.71658318304358e-06 | 6.70664798489389e-06 | 5.25051210775874e-06 | 7.45195400458137e-06 | 1.85788846995226e-06 | 9.18290147686821e-07 | 5.13156736766791e-06 | 1.30459372443255e-06 | 2.1367799335147e-06 | 1.36535314825349e-06 | 1.34561322289143e-06 | 4.89198567737074e-06 | 2.03369959074115e-06 | 0.42 | -1.3 | 0.044 | 1.91 | down |
| pos\_1150 | 1-Acetoxy-2-hydroxy-16-heptadecen-4-one | 0.00015 | 0.00026 | 0.00019 | 0.00021 | 0.00021 | 0.00024 | 0.00027 | 0.00027 | 0.00018 | 0.00028 | 0.00028 | 0.00031 | 0.00021 | 0.00027 | 1.27 | 0.35 | 0.037 | 2.01 | up |
| pos\_1180 | Ustiloxin B | 0.00038 | 0.00011 | 0.00032 | 0.00021 | 0.00033 | 3.22435747774862e-07 | 1.41789851109209e-06 | 1.23058559592231e-05 | 0.00013 | 8.73226451032093e-07 | 2.672021042301e-07 | 8.35321758639335e-07 | 0.00022 | 2.50351589518165e-05 | 0.11 | -4.9 | 0.018 | 2.30 | down |
| pos\_1248 | Cerebroside B | 2.09961928551289e-06 | 1.07254822091939e-06 | 1.91119367320607e-06 | 5.61763743287234e-06 | 7.27937688743303e-06 | 2.80738716624481e-06 | 1.09353927396022e-06 | 9.35717321816451e-07 | 0 | 0 | 8.4742868203384e-07 | 2.08240165138295e-06 | 3.46462711103142e-06 | 8.2651448819891e-07 | 0.24 | -8.8 | 0.045 | 2.04 | down |
| pos\_1249 | Quinquenoside III | 4.97797653238683e-06 | 3.6449387498287e-06 | 4.41150192560898e-06 | 5.72121314580859e-06 | 2.62968983836385e-06 | 5.63090614321353e-06 | 5.45775415226494e-06 | 8.55070703871328e-06 | 7.24801579382881e-06 | 6.67223761888877e-06 | 5.65853649207721e-06 | 5.15841637454096e-06 | 4.50270438920175e-06 | 6.45761124505233e-06 | 1.43 | 0.55 | 0.022 | 2.18 | up |

注：Fold\_change表示差异倍数，log2FC为差异倍数取log2的值，VIP为OPLS-DA模型的VIP值，P\_value为t检验的P值

差异代谢物筛选结果

1. H\_vs\_L.diff\_final.xls

常见问题

- OPLS-DA分析使用的是什么软件？能否进行多组分析？

  流程中目前使用的软件是R包ropls，该软件目前在进行OPLS-DA分析时限定只分析两组。具体可参考 https://bioconductor.org/packages/release/bioc/html/ropls.html。

- PCA分析和OPLS-DA分析优缺点？

  PCA分析可用于多组比较分析，且无需预先知道样品分组信息，能突出特异样本，但是对差异的解释性较差。OPLS-DA分析需预先给定样品分组信息，特别适合比较组间差异，可视化效果好，且能很好的挖掘出具体造成组间差异的代谢物。

- 差异代谢物太少如何处理？

  一般是由于数据本身的原因，需要检查样品间的相关性，同组内的相关性是不是比较差，也可以结合PCA图，可适当剔除样本后进行分析；另外OPLS-DA模型图中Q2值，该值比较小时也表明组间代谢物的差异不明显。此外，可以适当放宽筛选的阈值。

通过火山图（Volcano Plot）可以快速地查看代谢物在两个组中表达水平的差异，以及差异的统计学显著性。差异表达火山图如下：

- H\_vs\_L.volcano

图13 差异代谢物火山图

注：火山图中每个点代表一个代谢物，横坐标代表该组对比各物质的倍数变化（取以2为底的对数），纵坐标表示t检验的P-value（取以10为底的对数），散点大小代表OPLS-DA模型的VIP值，散点越大VIP值越大，筛选得到的差异表达代谢物越可靠。图中蓝色的点代表下调差异表达代谢物，红色的点代表上调差异表达代谢物，灰色代表检测到但差异不显著的代谢物。此外按P值排序后挑选前5个有定性的代谢物标注在图中

对筛选的差异代谢物进行聚类分析可进一步挖掘特征，其结果如下：

- H\_vs\_L\_diff\_heatmap

图14 差异代谢聚类热图

注：分析时进行了按样本归一化的处理

各组差异代谢物数目统计如下表：

表7 差异代谢物统计表

| group name | All diff | down-regulated | up-regulated |
| --- | --- | --- | --- |
| H\_vs\_L | 106 | 49 | 57 |

注：group name：差异代谢物分组信息；  
All diff：差异显著代谢物数目，up-regulated：上调代谢物的数目，down-regulated：下调代谢物数目。

基于韦恩图，可比较分析各组之间差异代谢物的关系，其结果如下图：

- only\_one\_group\_venn

图15 各组差异代谢物韦恩图

注：若超过5组，多个分组的只显示5元韦恩图，同时最多只考虑30个分组进行两两韦恩图绘制

##### 2.4.5 差异代谢物KEGG功能注释及富集分析

生物体中的复杂代谢反应及其调控并不单独进行，往往由不同基因和蛋白质形成复杂的通路和网络，它们的相互影响和相互调控最终导致代谢组发生系统性的改变。对这些代谢和调控通路的分析可以更全面，更系统的了解实验条件改变导致的生物学过程的改变，性状或疾病的发生机理和药物作用机制等生物学问题。

KEGG（Kyoto Encyclopedia of Genes and Genomes）数据库有助于研究者把基因、表达信息以及代谢物含量作为一个整体网络进行研究。作为有关Pathway的主要公共数据库，KEGG提供的整合代谢途径 (pathway)查询，包括碳水化合物、核苷、氨基酸等的代谢及有机物的生物降解，不仅提供了所有可能的代谢途径，而且对催化各步反应的酶进行了全面的注解，包含有氨基酸序列、PDB库的链接等等，是进行生物体内代谢分析、代谢网络研究的强有力工具。

差异代谢物在生物体内相互作用，形成不同的通路。利用KEGG数据库对差异代谢物进行注释，其中部分注释结果如下：

表8 差异代谢物KEGG功能注释部分结果展示

| ID | name | H5 | H4 | H2 | H6 | H1 | H3 | L1 | L5 | L4 | L2 | L6 | L3 | H\_Mean | L\_Mean | Fold\_change | log2FC | Pvalue | VIP | regulated | KEGG\_pathway\_annotation |
| --- | --- | --- | --- | --- | --- | --- | --- | --- | --- | --- | --- | --- | --- | --- | --- | --- | --- | --- | --- | --- | --- |
| pos\_1 | D-erythro-Dihydrosphingosine | 0.00017 | 0.00025 | 0.00028 | 0.0002 | 0.00014 | 0.00022 | 0.00013 | 0.00016 | 0.00013 | 0.00014 | 0.00014 | 0.00012 | 0.00021 | 0.00014 | 0.65 | -0.6 | 0.016 | 2.29 | down | Sphingolipid signaling pathway(ko04071);;Sphingolipid metabolism(ko00600);;Metabolic pathways(ko01100) |
| pos\_1073 | Neolinustatin | 8.65184137415296e-06 | 7.88622271234797e-06 | 9.85186204859414e-06 | 8.04562355504655e-06 | 5.96503890331222e-06 | 6.88259674573533e-06 | 1.27405048068724e-05 | 9.8469071217803e-06 | 8.4786020981128e-06 | 1.11270187582944e-05 | 9.08477593083803e-06 | 1.46742998177055e-05 | 7.88053088986486e-06 | 1.09920180889339e-05 | 1.39 | 0.47 | 0.023 | 2.29 | up | -- |
| pos\_108 | RHOIFOLIN | 9.23534477713109e-06 | 2.0370241796344e-06 | 1.82688113090293e-05 | 1.09351704987591e-05 | 3.78775372116092e-06 | 3.57014084103671e-05 | 1.71236608976507e-05 | 4.17430306845687e-05 | 3.84908284112322e-05 | 8.49582130333884e-05 | 6.2976908725647e-05 | 7.69590820373692e-05 | 1.33275854826803e-05 | 5.37086206316427e-05 | 4.03 | 2.41 | 0.01 | 2.52 | up | -- |
| pos\_1141 | Isovalerylglutamic acid | 2.03288102216096e-05 | 1.77626953626292e-05 | 2.5607171467383e-05 | 1.50831721446601e-05 | 1.72535810088484e-05 | 8.31919499750707e-06 | 3.6666681758369e-05 | 3.35311177847392e-05 | 2.53988822599635e-05 | 4.73858259215339e-05 | 3.1145888413144e-05 | 1.32588457165065e-05 | 1.73924375337729e-05 | 3.12312069757094e-05 | 1.80 | 0.82 | 0.031 | 2.17 | up | -- |
| pos\_1143 | PE(15:0/20:2(11Z,14Z)) | 6.36832831399463e-06 | 1.71658318304358e-06 | 6.70664798489389e-06 | 5.25051210775874e-06 | 7.45195400458137e-06 | 1.85788846995226e-06 | 9.18290147686821e-07 | 5.13156736766791e-06 | 1.30459372443255e-06 | 2.1367799335147e-06 | 1.36535314825349e-06 | 1.34561322289143e-06 | 4.89198567737074e-06 | 2.03369959074115e-06 | 0.42 | -1.3 | 0.044 | 1.91 | down | -- |
| pos\_1150 | 1-Acetoxy-2-hydroxy-16-heptadecen-4-one | 0.00015 | 0.00026 | 0.00019 | 0.00021 | 0.00021 | 0.00024 | 0.00027 | 0.00027 | 0.00018 | 0.00028 | 0.00028 | 0.00031 | 0.00021 | 0.00027 | 1.27 | 0.35 | 0.037 | 2.01 | up | -- |
| pos\_1180 | Ustiloxin B | 0.00038 | 0.00011 | 0.00032 | 0.00021 | 0.00033 | 3.22435747774862e-07 | 1.41789851109209e-06 | 1.23058559592231e-05 | 0.00013 | 8.73226451032093e-07 | 2.672021042301e-07 | 8.35321758639335e-07 | 0.00022 | 2.50351589518165e-05 | 0.11 | -4.9 | 0.018 | 2.30 | down | -- |
| pos\_1248 | Cerebroside B | 2.09961928551289e-06 | 1.07254822091939e-06 | 1.91119367320607e-06 | 5.61763743287234e-06 | 7.27937688743303e-06 | 2.80738716624481e-06 | 1.09353927396022e-06 | 9.35717321816451e-07 | 0 | 0 | 8.4742868203384e-07 | 2.08240165138295e-06 | 3.46462711103142e-06 | 8.2651448819891e-07 | 0.24 | -8.8 | 0.045 | 2.04 | down | -- |
| pos\_1249 | Quinquenoside III | 4.97797653238683e-06 | 3.6449387498287e-06 | 4.41150192560898e-06 | 5.72121314580859e-06 | 2.62968983836385e-06 | 5.63090614321353e-06 | 5.45775415226494e-06 | 8.55070703871328e-06 | 7.24801579382881e-06 | 6.67223761888877e-06 | 5.65853649207721e-06 | 5.15841637454096e-06 | 4.50270438920175e-06 | 6.45761124505233e-06 | 1.43 | 0.55 | 0.022 | 2.18 | up | -- |

注：KEGG\_pathway\_annotation为KEGG注释结果

差异代谢物KEGG功能注释结果

1. H\_vs\_L.KEGG\_diff\_anno.xls

HMDB数据库是一个全面的，高质量的代谢数据库，基于该数据库提供的代谢物分类信息对注释到的差异代谢进行统计绘图，其结果如下：

- H\_vs\_L\_metabolites\_classification\_top20

图16 各组差异代谢物HMDB分类图

注：该分类图只显示代谢分类数目按从大到小排序后的前20个

各个分组差异代谢物KEGG注释数目统计如下：

表9 各组差异代谢物KEGG注释结果统计

| Group | Diff\_num |
| --- | --- |
| H\_vs\_L | 106 |

注：Group为差异分组信息；Diff\_num为差异代谢物数目；Diff\_KEGG\_num为注释到KEGG的差异代谢物数目

差异代谢物KEGG功能注释通路图如下：

- ko00061
- ko00260
- ko00310
- ko00564
- ko00592
- ko00600
- ko00740
- ko00860
- ko01100
- ko02010
- ko04071
- ko04725
- ko04976
- ko05231

图17 各组差异代谢物KEGG注释结果图

注：红色为上调，绿色为下调

clusterProfiler是一个专门用于进行GO、KEGG富集分析R包。利用clusterProfiler选用超几何检验的方法对差异代谢物KEGG的注释结果进行富集分析，并绘制分类图、点图、网络图等三种图，下面是具体的结果。

富集分类结果图如下所示：

- H\_vs\_L\_KEGG\_pathway\_enrich\_barplot

图18 各组差异代谢物通路分类图

注：横轴为注释到该通路的差异代谢物数目，纵轴为通路名称

富集点图如下：

- H\_vs\_L\_KEGG\_pathway\_enrich\_dotplot

图19 差异代谢物KEGG富集图

注：横轴表示该通路中的差异代谢物与有通路注释的所有差异代谢物的比值

富集网络图如下：

- H\_vs\_L\_KEGG\_pathway\_enrich\_cnetplot

图20 差异代谢物KEGG富集网络图

注：图中淡黄色节点为通路，与之相连的小节点是注释到该通路的具体代谢物，颜色的深浅表示差异倍数取log2的值，该图最多显示5个通路

常见问题

- 代谢组可以和哪些组学联合分析辅助机理研究？

  目前可与转录组、蛋白组、微生物等进行联合分析。其中与转录组、蛋白组联合分析内容包括KEGG通路分析、共富集分析、差异代谢物和基因的趋势分析、相关性网络、典型相关分析等；与微生物可进行代谢物与OTU相关性分析、相关性网络分析、协惯量分析等。

- 转录组和代谢组生物学重复数量不同，是否可以做联合分析？

  可以做，一般是代谢组样品多于转录组样品，所以目前预设两种方式：一是直接挑选代谢样品与转录组样品对应；二是多个代谢组样品均值合并后与转录组样品对应。这两种方式下可以直接让软件自动对应，也可以通过填写文件进行设置样品对应关系。

#### 2.5 附录：英文版实验方法

##### 2.5.1 Metabolites Extraction

The LC/MS system for metabolomics analysis is composed of Waters Acquity I-Class PLUS ultra-high performance liquid tandem Waters Xevo G2-XS QTof high resolution mass spectrometer. The column used is purchased from Waters Acquity UPLC HSS T3 column (1.8um 2.1\*100mm)

Positive ion mode: mobile phase A: 0.1% formic acid aqueous solution; mobile phase B: 0.1% formic acid acetonitrile

Negative ion mode: mobile phase A: 0.1% formic acid aqueous solution; mobile phase B: 0.1% formic acid acetonitrile

Injection volume 1μL

##### 2.5.2 LC-MS/MS Analysis

Waters Xevo G2-XS QTOF high resolution mass spectrometer can collect primary and secondary mass spectrometry data in MSe mode under the control of the acquisition software (MassLynx V4.2, Waters). In each data acquisition cycle, dual-channel data acquisition can be performed on both low collision energy and high collision energy at the same time. The low collision energy is 2V, the high collision energy range is 10~40V, and the scanning frequency is 0.2 seconds for a mass spectrum. The parameters of the ESI ion source are as follows: Capillary voltage: 2000V (positive ion mode) or -1500V (negative ion mode); cone voltage: 30V; ion source temperature: 150°C; desolvent gas temperature 500°C; backflush gas flow rate: 50L/ h; Desolventizing gas flow rate: 800L/h.

##### 2.5.3 Data preprocessing and annotation

The raw data collected using MassLynx V4.2 is processed by Progenesis QI software for peak extraction, peak alignment and other data processing operations, based on the Progenesis QI software online METLIN database and Biomark’s self-built library for identification, and at the same time, theoretical fragment identification and mass deviation All are within 100ppm.

表10 代谢组产品

| 产品名称 | 测序类型 | 定量方法 |
| --- | --- | --- |
| 普通非靶（LC/GC） | GC-TOF/LC-QTOF/QE | 相对定量 |
| 顶空进样非靶标 | GC/MS | 相对定量 |
| 脂质组非靶标 | LC-QTOF | 相对定量 |
| 肠道菌群非靶标 | GC-TOF/LC-QTOF/QE | 相对定量 |
| 靶标代谢组 | LC-QQQ/GC-Q | 绝对定量 |
| 动物高通量靶标 | LC-MS/MS | 相对定量 |
| 植物高通量靶标 | LC-MS/MS | 相对定量 |
| 花青素高通量靶标 | LC-MS/MS | 相对定量 |
| 氨基酸kit | LC-QQQ | 绝对定量 |
| 短链脂肪酸kit | GC-MS | 绝对定量 |
| 游离脂肪酸kit | GC-MS | 绝对定量 |
| 神经递质kit | LC-QQQ | 绝对定量 |
| 胆汁酸kit | LC-QQQ | 绝对定量 |

结果目录说明

### 3 参考文献

1. Chen, W., Gong, L., Guo, Z., et al., A Novel Integrated Method for Large-Scale Detection, Identification, and Quantification of Widely Targeted Metabolites: Application in the Study of Rice Metabolomics. Molecular Plant, 2013, 6(6):1769-1780.
2. Fraga C.G., Clowers B.H., Moore R.J., et al., Signature-discovery approach for sample matching of a nerve-agent precursor using liquid chromatography-mass spectrometry, XCMS, and chemometrics. Anal Chem, 2010. 82(10): p. 4165-73.
3. Garcia A, Barbas C.Garcia A, Barbas C. Gas chromatography-mass spectrometry (GC-MS)-based metabolomics[M]//Metabolic Profiling. Humana Press, 2011: 191-204.. Humana Press, 2011: 191-204.
4. Chen, Y., et al., RRLC-MS/MS-based metabonomics combined with in-depth analysis of metabolic correlation network: finding potential biomarkers for breast cancer. Analyst, 2009.134(10): p. 2003-11.
5. Thévenot E A, Roux A, Xu Y, et al. Analysis of the Human Adult Urinary Metabolome Variations with Age, Body Mass Index, and Gender by Implementing a Comprehensive Workflow for Univariate and OPLS Statistical Analyses. Journal of Proteome Research, 2015, 14(8):3322-35.
6. Yu G, Wang L, Han Y, He Q (2012). clusterProfiler: an R package for comparing biological themes among gene clusters. OMICS: A Journal of Integrative Biology, 16(5), 284-287.
7. Chong J, Xia J. MetaboAnalystR: an R package for flexible and reproducible analysis of metabolomics data[J]. Bioinformatics, 2018, 34(24): 4313-4314.

- 摘要
- 1 代谢组主要分析内容
- 2 非靶向代谢组分析结果
  - 2.1 实验流程
    - 2.1.1 实验试剂与仪器
    - 2.1.2 样品提取
    - 2.1.3 上机检测
    - 2.1.4 代谢物定量
  - 2.2 数据评估
    - 2.2.1 主成分分析（PCA）
    - 2.2.2 聚类热图分析
    - 2.2.3 重复相关性评估
  - 2.3 代谢物注释
    - 2.3.1 KEGG数据库注释
    - 2.3.2 HMDB数据库注释
    - 2.3.3 Lipidmaps数据库注释
  - 2.4 样本分组数据分析
    - 2.4.1 各组主成分分析
    - 2.4.2 差异倍数分析
    - 2.4.3 差异分组的正交偏最小二乘法判别分析（OPLS-DA）
    - 2.4.4 差异代谢物筛选
    - 2.4.5 差异代谢物KEGG功能注释及富集分析
  - 2.5 附录：英文版实验方法
    - 2.5.1 Metabolites Extraction
    - 2.5.2 LC-MS/MS Analysis
    - 2.5.3 Data preprocessing and annotation
- 3 参考文献

Copyright © 2009-2021 北京百迈客生物科技有限公司版权所有 京ICP备10042835号

公司地址：北京市顺义区南法信府前街12号顺捷大厦5层

- Tel:400-600-3186
- Fax:010-57045001
- E-mail:tech@biomarker.com.cn
- 微信:biomarker\_tech
- 百迈客生物云平台
- 关于我们
